# Efficacy of Intranasal Esketamine in Treatment‐Resistant Depression: A Six‐Month Real‐World Follow‐Up Study of Depressive Symptoms, Hopelessness, and Suicide Risk

**DOI:** 10.1002/hup.70008

**Published:** 2025-07-09

**Authors:** Maurizio Pompili, Maria Anna Trocchia, Ludovica Longhini, Eva Dispenza, Cristina Di Legge, Salvatore Sarubbi, Denise Erbuto, Isabella Berardelli

**Affiliations:** ^1^ Department of Neurosciences Mental Health and Sensory Organs Suicide Prevention Centre Sant'Andrea Hospital Sapienza University of Rome Rome Italy; ^2^ Department of Human Neurosciences Sapienza University of Rome Rome Italy; ^3^ Psychiatry Residency Training Program Faculty of Medicine and Psychology Sapienza University of Rome Psychiatry Unit Sant'Andrea Hospital Rome Italy

**Keywords:** esketamine, suicide prevention, suicide risk, treatment‐resistant depression

## Abstract

**Objective:**

Treatment‐resistant depression is one of the most significant clinical challenges in psychiatric practice. The primary aim of the present study was to assess the efficacy and tolerability of intranasal esketamine on depressive symptoms in a real‐world outpatient setting. A secondary objective was to explore the potential benefits of intranasal esketamine on hopelessness and suicide risk (suicidal ideation and suicide attempts).

**Methods:**

Twenty‐one patients diagnosed with treatment‐resistant depression were treated with intranasal esketamine. Depressive symptoms (MADRS and BDI), suicide risk (C‐SSRS), and hopelessness (BHS) were assessed. We conducted a mixed model for repeated measures analysis to evaluate changes from baseline (T0), 3‐month follow‐up (T1), and 6‐month follow‐up (T2).

**Results:**

Results indicated that depressive symptoms decreased over time. Specifically, both clinician and self‐report measures show lower levels of depressive symptoms at 3‐month and 6‐month follow‐up. We also found a significant decrease in the presence of suicidal ideation between T0 and T2. Finally, patients also reported a reduction in hopelessness levels over time.

**Conclusions:**

Our findings indicate an overall response regarding depressive symptoms, hopelessness, and suicidal ideation after esketamine in treatment‐resistant depression at 3‐month and 6‐month follow‐up assessments.

## 1 Introduction

Nearly 33% of individuals affected by major depressive disorder (MDD) do not have complete remission of symptoms with currently available treatment options, paving the way to treatment‐resistant depression (TRD) (Rush et al. 2006; McIntyre et al. 2023). Approximately 30% of patients with TRD attempt suicide at least once in their lifetime (Hantouche et al. 2010). Given the high suicide risk of TRD patients, it is of paramount importance to investigate whether specific treatments might impact suicide risk. Moreover, in TRD, depressive symptoms lead to a loss of quality of life, more hospitalizations, decreases in productivity, and higher healthcare costs (McIntyre et al. 2023). According to a recent cohort study (Pompili, Dell'Osso, et al. 2023), the management and treatment of major depression are particularly complex, and there is a lack of rapid and effective treatment options to reduce depressive symptoms in the presence of actual suicide risk.

Ketamine is a racemic compound with two enantiomers: S‐ketamine (esketamine) and R‐ketamine. Esketamine is the S‐enantiomer of ketamine, exhibiting more potency at the NMDA glutamate receptor than its R‐ketamine counterpart. This drug has been extensively utilized for numerous years as an intravenous anesthetic; over the past two decades, it has garnered significant interest in psychiatry due to its established fast antidepressant and anti‐suicidal properties (Zarate et al. 2006; Berman et al. 2000; Diazgranados et al. 2010; Wilkinson et al. 2018; Loo et al. 2023). Esketamine is a non‐competitive antagonist on NMDA receptors located on GABAergic inhibitory interneurons that suppress glutamate release from downstream glutamatergic neurons (Krystal et al. 2019). GABA interneurons are nerve cells that are inhibitory in function. Esketamine‐mediated inhibition of these GABA interneurons could result in enhanced release of glutamate and hence greater action of glutamate on AMPA receptors (Murrough et al. 2017). S‐ketamine may exert its antidepressant effects by indirectly augmenting glutamatergic transmission by reducing synaptic GABAergic inhibition (Widman and McMahon 2018; Gerhard et al. 2020). This pathway synthesizes and releases enhanced brain‐derived neurotrophic factor (BDNF) (Zhou et al. 2014). Subsequently, esketamine had antidepressant‐like effects and promoted synaptogenesis through the ERK and mTORC1 signaling pathways (Ignácio et al. 2016; Mendoza et al. 2011). Esketamine has demonstrated efficacy in treatment‐resistant depression (TRD) and has accumulated significant attention in MDD for its application in psychiatric emergencies involving suicidal ideation with intent. Concerning TRD, randomized controlled trials (RCTs) have demonstrated that intranasal esketamine is both safe and effective when used in conjunction with oral antidepressants, resulting in a swift alleviation of depressive symptoms (Daly et al. 2018; Fedgchin et al. 2019; Popova et al. 2019). Intranasal esketamine is indicated for adults diagnosed with treatment‐resistant depression (TRD) and for the rapid reduction of symptoms of major depressive disorder in patients with acute suicidal ideation or behavior, a feature also representing a psychiatric emergency (Please refer to the summary of product characteristics for full indications, details, and type of depressive episode in major depressive disorder as well as for any kind of psychiatric emergency.). Such features were investigated through the ASPIRE studies trials (Fu et al. 2020; Ionescu et al. 2021) which demonstrated that esketamine plus a comprehensive standard of care (SOC) rapidly reduced depressive symptoms in MDD patients with acute suicidal ideation or behavior, especially in those with a history of suicide attempts, providing a new treatment option for this particularly ill and vulnerable population.

The ASPIRE studies had a primary endpoint investigating the change from baseline (Day 1, pre‐dose) to 24 h post‐first dose in depressive symptoms (measured by MADRS total score). There was also a key secondary endpoint investigating the change from baseline (Day 1, pre‐dose) to 24 h post‐first dose in severity of suicidality (measured by CGI‐SS‐r).

The authors found that 24 hours after the first dose of the study medication, esketamine plus SOC demonstrated a clinically meaningful and statistically significant reduction of depressive symptoms. However, both treatment groups (placebo + SOC and esketamine + SOC) experienced an improvement in the severity of suicidality from baseline to 24 h after the first dose. However, the difference between treatment groups was not statistically significant, possibly due to various factors, such as hospitalization and implemented medical attention with an empathic understanding of the suicidal crisis (Pompili 2019, 2024). As for the severity of depression, intranasal esketamine + SOC improved depressive symptoms throughout the treatment phase.

Clinical trials and real‐world experiences associated with esketamine offer insights into esketamine's therapeutic efficacy, safety profile, and implications for clinical practice in patients with TRD (Song et al. 2024). In the SUSTAIN‐1 study, intranasal esketamine plus an SSRI/SNRI antidepressant resulted in being more effective than placebo nasal spray plus an SSRI/SNRI antidepressant in delaying relapse in TRD patients who had been previously stabilized (up to achieving stable remission) with esketamine nasal spray plus an antidepressant (Daly et al. 2018).

In SUSTAIN‐2, flexible doses of intranasal esketamine along with SSRI/SNRI antidepressants were given continuously in an open‐label setting for up to 1 year, showing good safety and tolerability over the medium to long term (Wajs et al. 2020). Finally, SUSTAIN‐3, a global multicenter study, evaluated the efficacy and long‐term safety of weekly or biweekly intranasal esketamine, combined with an oral antidepressant, in patients with TRD (Zaki et al. 2023). Moreover, the efficacy and safety of intranasal esketamine in TRD patients were also investigated in three 4‐week Randomized Controlled Trials: Studies TRANSFORM‐1 and TRANSFORM‐2 in adults aged 18 to 65 (Fedgchin et al. 2019; Popova et al. 2019) and TRANSFORM‐3 (Ochs‐Ross et al. 2020; Pompili, Sarli, et al. 2023).

Based on the efficacy of esketamine in treating TRD, in this prospective study, we aimed to evaluate the effectiveness of intranasal esketamine on depressive symptoms in a clinical sample of patients with TRD. Evaluations were undertaken at the baseline and 3 months and 6 months after initiating esketamine treatment. The study aimed to offer ideas about the clinical application of esketamine as a treatment for TRD. A secondary aim was to evaluate the role of esketamine in reducing suicide risk and hopelessness in patients with TRD. Finally, we evaluated the presence of esketamine‐related side effects over the three time points.

## 2 Methods

This was an observational prospective study comprising a total of 21 patients with TRD (12 females, 9 males), with a mean age of 49.71 years (SD = 17.00) who were treated with intranasal esketamine in compliance with indications provided by the Italian regulatory agency for drugs (Agenzia Italiana del Farmaco; AIFA). Two expert psychiatrists made psychiatric diagnoses using the Diagnostic and Statistical Manual of Mental Disorders, Fifth Edition (DSM‐5) criteria (American Psychiatric Association 2013). For the identification of TRD, we considered the failure to respond to at least two different antidepressant treatments of adequate dose and duration, despite appropriate adherence.

Eligibility criteria for patients were as follows: over 18 years of age and a diagnosis of TRD. For the definition of TRD following DSM‐5, we adopted the one proposed by McIntyre et al. (2023) considered TRD when there was “failure to respond to two or more antidepressant regimens despite adequate dose and duration and adherence to treatment.” A qualified psychiatrist considered the previous treatment's dose, duration, and adherence. Patients who had previously been treated with an SSRI or SNRI, which could still be ongoing, were subsequently treated with intranasal esketamine (plus an oral antidepressant) according to the summary of product characteristics.

Patients with comorbid organic pathologies (i.e., untreated vascular hypertension or previous cerebrovascular disorders) that represented an absolute contraindication to esketamine according to the summary of product characteristics were excluded.

All participants received a comprehensive explanation of the study procedures and signed a written informed consent. The local ethics committee approved the study (Protocol Number: RIF. CE 6935/2022). All patient data were treated confidentially and anonymously, and the study was conducted in line with the Helsinki Declaration (World Medical Association 2013).

### 2.1 Study Procedures and Measurements

Esketamine is intended to be self‐administered by the patient under the direct supervision of a healthcare provider (physician and nurse). A treatment session involves nasal administration of esketamine and a period of post‐administration. The administration of esketamine and the post‐observation period were performed in an appropriate clinical setting.

We administered intranasal esketamine in accordance with the summary of product characteristics, which included a blood pressure assessment prior to administration. We measured blood pressure before administration, at 40 min post‐administration and at the end of the observation period. The outpatient treatment for each patient was weeks 1–4: induction phase (initial dose for day 1: 56 mg, subsequent doses: 56 mg or 84 mg twice per week). Weeks 5–8: Maintenance phase (56 mg or 84 mg once a week). From week 9: 56 mg or 84 mg every 2 weeks or once a week.

The following psychometric tools were administered: the Montgomery–Asberg Depression Rating Scale (MADRS; Montgomery and Asberg 1979) and the Beck Depression Inventory‐II (BDI‐II; Beck et al. 1996), to assess depressive symptoms. The Columbia‐Suicide Severity Rating Scale (C‐SRSS; Posner et al. 2011) assesses suicidal ideation and behaviors, and the Beck Hopelessness Scale (BHS; Beck et al. 1974) evaluates hopelessness about the future. Psychometric assessments were collected before the administration of esketamine at baseline (T_0_), 3 months (T_1_), and 6 months (T_2_) after treatment began by two trained psychiatrists.

**The Montgomery‐Asberg Depression Rating Scale** is a clinician instrument used to assess the severity of depression symptoms among patients with a diagnosis of MDD. It is designed to assess changes resulting from antidepressant therapy in the previous week.

**The Beck Depression Inventory‐II** is a 21‐item self‐report instrument that evaluates the severity of depressive symptoms during the previous 2 weeks. Each item is scored from 0 to 3 to assess the severity of the symptom.

**The Columbia‐Suicide Severity Rating Scale** is a semi‐structured interview to assess both suicidal ideation and suicide attempts in individuals aged 12 years and older. The first section evaluates the severity of suicidal ideation, ranging from a wish to be dead to active suicidal ideation with a specific plan and intent. The second section evaluates the intensity of suicidal ideation (i.e., frequency, duration, controllability, deterrents, and reasons for ideation). The score was derived from the sum of these items (ranging from 0 to 25). The third section evaluates suicidal behaviors. According to the protocol of this instrument, we assessed suicide risk over the last month and suicide attempts over the last 3 months using the Lifetime version at the baseline stage (T0) and the Since Last Visit version at the follow‐up stages (T1 and T2).

**The Beck Hopelessness Scale** is a 20‐item self‐report measure of hopelessness about the future. Several international studies reported good psychometric properties of the BHS and suggested satisfactory ability in predicting subsequent suicidal behavior and general health and social functioning (Beck et al. 1990; Pompili et al. 2013).

### 2.2 Statistical Analysis

Statistical analyses were performed with the Statistical Package for Social Sciences (SPSS 29.0, IBM Corp, Armonk, NY). Numbers and percentages were reported for categorical variables, while mean and standard deviation (SD) were reported for continuous variables. For dichotomous variables with repeated measurements (i.e., presence/absence of side effects and suicidal ideation), the Cochran's Q test was used to compare the proportion of patients experiencing events at three time points (T0, T1, T2), followed by pairwise comparisons. Furthermore, analyses for continuous variables (MADRS, BHS, BDI, and C‐SSRS Intensity) were conducted using Mixed Models with Repeated Measures (MMRM). Each model included time as a fixed effect, while subjects were treated as random effects.

A first‐order autoregressive covariance structure [AR (1)] was specified to account for the correlation between repeated measurements taken at three equidistant points (baseline, 3 months, and 6 months). This structure assumes that the more temporally close observations are more correlated with each other, with the correlation decreasing exponentially as the time interval between assessments increases.

Estimates were obtained using the Restricted Maximum Likelihood method (REML). The estimated marginal averages were compared between different time points, with the first time (T_0_) as the reference category. The analyses followed a modified intention‐to‐treat (mITT) approach, including all participants who had at least one post‐baseline assessment. This is consistent with the assumptions of MMRM, which handles missing data based on the missing at random (MAR) assumption. Due to multiple comparisons, Bonferroni correction was applied to control for type I errors. All results were reported with 95% confidence intervals. Lastly, to explore potential differences in baseline characteristics between participants who completed the 6‐month follow‐up and those who discontinued the study, comparison analyses were conducted using non‐parametric tests (Mann‐Whitney for continuous variables and Fisher's exact test for categorical variables). The statistical significance level was set at *p* < 0.05.

### 2.3 Results

Characteristics of the sample are reported in Table 1. At baseline, 11 patients (52.4%) reported the presence of some degree of suicidal ideation, and two patients (9.5%) attempted suicide in the previous 3 months. The dosage of esketamine at baseline was 56 mg.

**TABLE 1:** Characteristics of the sample.

Variables	N (%); M ± SD
Sex (female)	12 (57.1)
Age	49.71 ± 17.00
Marital status	
Single	10 (47.6)
Married	8 (38.1)
Divorced	3 (14.3)
Job condition	
Employee	12 (57.1)
Unemployed	5 (23.8)
Retired	4 (19.0)
Baseline rates	
CSSRS ‐ suicidal ideation (yes)	11 (52.4)
CSSRS ‐ suicidal ideation intensity	5.10 ± 5.78
CSSRS ‐ suicide attempt (yes)	2 (9.5)
MADRS	33.38 ± 4.67
BDI	42.62 ± 8.03
BHS	16.62 ± 2.87
Baseline AD treatments	
SSRIs	13 (61.9)
SNRIs	8 (38.1)
Other AD	5 (23.8)

During the first treatment, 13 patients (61.9%) experienced at least one side effect, which was generally solved in the first 40 min (Table 2).

**TABLE 2:** Side Effects experienced by patients during esketamine treatment.

	N (%)
Side effects (yes)	13 (61.9)
Dissociative symptoms	6 (28.6)
Anxiety	5 (23.8)
Dysphoria	4 (19.0)
Sleepiness	4 (19.0)
Cardiovascular symptoms	3 (14.3)
Dizziness	2 (9.5)
Insomnia	2 (9.5)
Gastrointestinal symptoms	1 (4.8)
Hypoglycemia	1 (4.8)

Changes in all clinical variables over time are reported in Tables 3 and 4.

**TABLE 3:** Mixed model for repeated measures.

Variables	Test	Baseline T_0_ Mean (SE)	Mean change (SE)		Adj. Pairwise comparisons 95% CI	
			3 months T_1_	6 months T_2_	T_0_–T_1_	T_0_–T_2_
MADRS	*F* = 65.50^***^	33.38 (1.22)	−11.95 (1.41)	−19.50 (1.82)	−15.26/−8.65	−23.70/−15.30
BDI	*F* = 25.41^***^	42.62 (2.21)	−13.43 (2.19)	−19.28 (3.01)	−18.57/−8.28	−26.24/−12.31
C‐SSRS intensity	*F* = 3.06	5.10 (1.13)	−2.14 (0.98)	−3.09 (1.40)	−4.46/0.17	−6.34/0.16
BHS	*F* = 14.31^***^	16.62 (0.76)	−3.86 (0.76)	−4.31 (1.04)	−5.63/−2.08	−6.71/‐ 1.90

Abbreviation: Adj = Adjustment for Bonferroni Correction.

*** *p* < 0.001.

**TABLE 4:** Cochrane's Q test.

Variables	Time 0	Time 1	Time 2	Test	Adj. Pairwise comparisons	
					T_0_–T_1_	T_0_–T_2_
Presence of suicidal ideation	11 (52.4%)	7 (33.3%)	2 (14.3%)	*Q*_*2*_ = 11.14^**^	0.357, *p* = 0.062	0.500, *p* = 0.004
Presence of side effects	13 (61.6%)	7 (33.3%)	5 (35.7%)	*Q*_*2*_ = 6.50^*^	0.286, *p* = 0.043	0.214, *p* = 0.199

Abbreviation: Adj = Adjustment for Bonferroni Correction.

* *p* < 0.05.

** *p* < 0.001.

Depressive symptoms decreased for both clinician and self‐report measures during the three time points. For clinician's evaluations, the model was statistically significant (*F* = 63.50, *p* < 0.001), indicating that MADRS scores progressively decreased in depressive symptoms over time [T_0_ *M* = 33.38 (SE = 1.22), T_1_ *M* = 21.43 (SE = 1.22), T_2_ *M* = 13.88 (SE = 1.47)]. Pairwise comparisons with Bonferroni correction showed a significant reduction in MADRS scores from T_0_ to T_1_ (*p*_adj_ < 0.001), and from T_0_ to T_2_ (*p*_adj_ < 0.001). Similarly, the result for BDI scores showed significant changes over time [*F* = 25.41, *p* < 0.001; T_0_ *M* = 42.62 (SE = 2.21), T_1_ *M* = 29.19 (SE = 2.21), T_2_ *M* = 23.34 (SE = 2.54)]. Adjusted pairwise comparisons showed significant differences between T_0_ and T_1_ (*p*_adj_ < 0.001), and between T_0_ and T_2_ (*p*_adj_ < 0.001), indicating a reduction in depressive symptoms over time.

Cochran's Q Test indicated a significant difference across the three times for the presence of suicidal ideation (*Q*_2_ = 11.14, *p* = 0.004), pairwise comparison showed an important difference between T_0_ and T_2_ (*p*_adj_ = 0.004), and a marginal difference between T_0_ and T_1_ (*p*_adj_ = 0.062). No difference was found between T_1_ and T_2_. Instead, according to suicidal ideation intensity, the model showed a trend toward significance (*F* = 3.06, *p* = 0.060), suggesting a possible change in suicidal ideation intensity across the three time points. Although the reduction was not significant, the trend suggests a clinical decrease that may be investigated in studies with larger sample sizes.

Regarding hopelessness, the model showed a significant effect of the time (*F* = 14.31, *p* < 0.001), indicating a meaningful change in BHS scores across the three‐point time [T_0_ *M* = 16.62 (SE = 0.76), T_1_ *M* = 12.76 (SE = 0.76), T_2_ *M* = 12.31 (SE = 0.89)]. Pairwise comparisons confirmed that both T_1_ (*p* < 0.001) and T_2_ (*p* < 0.001) scores were lower than T_0_, demonstrating a reduction in the levels of hopelessness over time.

Lastly, according to the presence of side effects, Cochran's Q Test indicated a significant difference across the three times (*Q*_2_ = 6.50, *p* = 0.039); post hoc pairwise comparison showed an important difference between T_0_ and T_1_ (*p*_adj_ = 0.043), while no significant differences were found between T_0_ and T_2_ (*p*_adj_ = 0.199), and between T_1_ and T_2_ (*p*_adj_ = 1.00).

**FIGURE 1:**
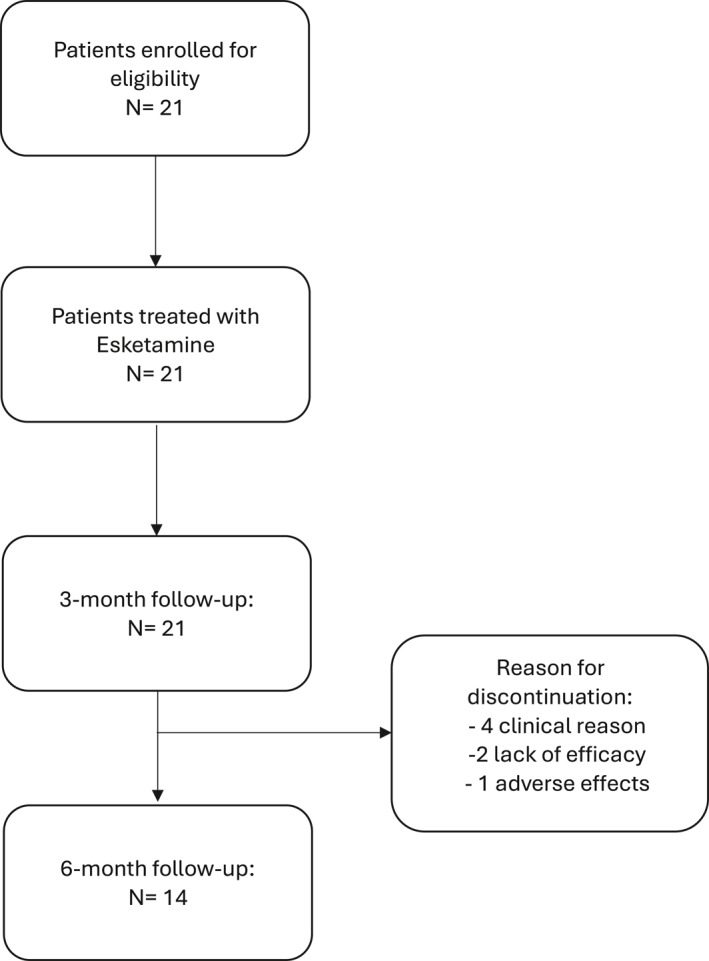
CONSORT‐style flow diagram of participant progression through the study.

During the study, seven patients discontinued the treatment (Figure 1). All patients discontinued in the time between 3 months (T_1_) and 6 months (T_2_), four patients for clinical reasons, two patients due to the lack of perceived benefit, and one patient for severe side effects (dissociative symptoms during the treatment). Results to explore potential differences between patients who completed the 6‐month follow‐up and patients who discontinued the treatment revealed no statistically significant differences. Specifically, no differences were found for age (*U* = 33.00, *p* = 0.232), sex (Fisher *p* = 0.397), MADRS score (*U* = 37.00, *p* = 0.369), BDI score (*U* = 41.50, *p* = 0.575), BHS score (*U* = 45.00, *p* = 0.761), presence of side effect (Fisher *p* = 0.656), suicidal ideation (Fisher *p* = 0.183) and suicide attempt (Fisher *p* = 0.533); indicating no significant baseline differences between completers and dropouts.

## 3 Discussion

The results of this prospective study, which was comprised of 21 outpatients with TRD, showed that depressive symptoms assessed with both clinician and self‐report measures decreased over time (3‐month and 6‐month follow‐up) compared to baseline (T_0_), where the mean score (MADRS = 33.38 ± 4.67) was in line with registration trials (Popova et al. 2019; Daly et al. 2018).

These results on depressive outcomes confirm the efficacy of intranasal esketamine on depressive dimensions (McIntyre et al. 2021). A meta‐analysis of five trials demonstrated the efficacy of intranasal esketamine augmentation in treating major depressive disorder (MDD) (Papakostas et al. 2020).

Our study also focused on the efficacy of esketamine on suicide risk in patients with TRD. Patients experiencing such resistance to treatment represent a subpopulation of depressed patients with extra burden due to persistence of symptoms, lower quality of life, loss of faith in treatment, and greater caregivers' dependence. Suicide risk may stem from the combination of such conditions and the suffering related to mental pain (Pompili et al. 2022).

The results showed that, although at baseline, 11 patients reported some level of suicidal ideation, at T_2_, two patients presented suicidal ideation. The presence of suicidal ideation decreased from baseline to follow‐up at 6 months. Although a reduction was observed over time in terms of presence/absence, statistical analysis showed a partial effect for the intensity score, showing only a significant trend. Therefore, any conclusions about the impact of esketamine on the intensity of suicidal ideation must be cautiously interpreted. The small sample size may have limited the study's statistical power, and future studies with larger samples will be required to clarify this possible effect.

Finally, patients also reported lower scores in BHS; specifically, hopelessness levels decreased from T_0_ to T_2_.

Several studies focused on the fundamental role of hopelessness in suicide risk (Beck et al. 1985; Beck 1986; Dahlsgaard et al. 1998; Beck et al. 1999).

Our findings support the efficacy of esketamine on hopelessness and suggest a possible role of this treatment in the amelioration of depressive symptoms that impact trusting the future and may pave the way to suicide risk. Canuso et al. (2018) conducted a double‐blind proof‐of‐concept study to examine the potential effect of esketamine, administered with an intranasal device, on suicidal ideation in patients with major depressive disorder who are at imminent risk for suicide. Their preliminary finding indicated that intranasal esketamine, given in addition to comprehensive standard‐of‐care treatment, may result in significantly rapid improvement in depressive symptoms, including some measures of suicidal ideation (Canuso et al. 2018). Later, results from the ASPIRE I and ASPIRE II phase III double‐blind clinical trials showed that intranasal esketamine plus the comprehensive standard of care was more effective than placebo nasal spray plus the comprehensive standard of care in rapidly reducing depressive symptoms (as measured by the MADRS scale at 24 h after a single dose of esketamine, the primary endpoint) in patients with MDD and actual risk of suicide. However, the two twin studies failed in achieving the key secondary endpoint: even if the patients experienced a rapid and clinically meaningful reduction in the severity of suicidality (as measured by the CGI‐SS‐r at 24 h after the first dose), this reduction was not statistically different from the control group. This may be partly due to the benefit of hospitalization, the intensive clinical contact patients received as study participants, and/or the method used to assess suicidality (Canuso et al. 2021). It is worth noting that such variables could have also played a role in our sample. The administration of esketamine consisted of sessions with more than one doctor involved, a nurse, and a psychologist, which could have provided intensive clinical care capable of influencing suicide risk and future expectation as measured by the hopelessness scale.

Major depression and hopelessness are among the most studied risk factors for suicide and are consistently featured in risk factor guidelines developed by major national and international organizations. An essential aspect of this study is that patients also reported a lower score in BHS; specifically, hopelessness levels decreased from T_0_ to T_2_. Beck et al. (1974) identified hopelessness as the link between depression and suicide, suggesting that may be more relevant than depressive symptoms in investigating suicide risk. Recent papers continued the exploration of hopelessness as a cognitive risk factor involved in suicide risk (Berardelli et al. 2023). Thus, the effect of esketamine on reducing hopelessness levels could be a promising result in suicide risk prevention. Furthermore, such results are relevant because they shed light on the complexity of suicide risk, with the need to pay attention not only to diagnosis but also to the phenomenology of suicide risk in major depression (Pompili 2019, 2020, 2022).

Although intranasal esketamine is recommended for the rapid reduction of symptoms of major depressive disorder in patients with a psychiatric emergency, our findings indicate an effect on suicidal ideation also for such an emergency when treating patients with TRD. The effect of esketamine treatment on suicidal ideation could involve several additional factors, including a strong therapeutic alliance and the high level of assistance of patients treated with esketamine. We acknowledge that therapeutic alliance has a beneficial effect on treatment‐related change in suicide ideation in patients with TRD, as suggested in previous literature (Fartacek et al. 2023).

One aspect that deserves attention in our sample is related to the number of patients who discontinued the treatment (7 out of 21). The reason for such an event was often associated with the logistical problems in reaching our hospital. The attendance of regular sessions could not be guaranteed by patients who did not rely on caregivers, had difficulties in organizing their work, or had problems combining various duties on their own.

Finally, regarding side effects, only one patient discontinued esketamine treatment for the presence of severe side effects (severe dissociation and hyperthension). Results indicated a significant difference across the time points, showing a significant decrease between T0 and T1. Furthermore, no patients required dose adjustment of esketamine due to side effects. Results demonstrated that in our sample, the most frequent adverse effects under treatment with esketamine were nausea, dissociative symptoms, anxiety, and sedation. Patients with dissociative symptoms generally peaked at 40 minutes after Esketamine administration and resolved in 1.5 h. These results align with previous studies that evaluated the long‐term safety of esketamine (Yang et al. 2022; Zhang et al. 2023).

The present study has several limitations; first, the small number of patients limits the generalizations based on the results. Second, there is no control population, and the follow‐up period is 6 months, which warrants a longer observation period. Third, although we used a precise definition of TRD, this may not necessarily apply to other populations, making it difficult to compare. Furthermore, due to the small sample size, the study is limited in the generalizability of results related to suicide risk and hopelessness. Particularly, the ability to detect small effects is weak, and subgroup analysis between patients who had suicidal ideation at baseline and those who did not remains impossible due to the sample size.

Our study also has the strength of follow‐up observation of patients treated with esketamine for unique variables such as suicide risk and hopelessness. While suicide risk has been explored in patients with major depressive disorder treated with esketamine, it is of note that TRD patients with suicide risk have seldom been investigated. Of further interest is the investigation of hopelessness, which is often associated with depressive symptoms and it is a proxy both for assessing suicide risk and quality of life.

## Author Contributions

Conceptualization: M.P. and I.B., Data acquisition: M.A.T., L.L., E.D., and C.D.L., Statistical analysis: S.S., Drafting of the manuscript: M.P. and I.B, Reviewing and editing: M.P., D.E., and I.B. All authors reviewed the manuscript, revised it critically for important intellectual content, have read and approved the final submitted manuscript, and agree to be accountable for the work contained within.

## Consent

All named co‐authors consent to publication. All authors have accepted responsibility for the entire content of this manuscript and consented to its submission to the journal.

## Conflicts of Interest

Prof. M. Pompili wishes to disclose that in the last 5 years, he has received lectures and advisory board honoraria or has engaged in clinical trial activities with Angelini Pharma, Allergan, Johnson & Johnson, Lundbeck, Merck Sharp and Dohme, Otsuka, Rovi, Italfarmaco, Pfizer Inc., Fidia, Viatris, Recordati, Boehringer Ingelheim, Neopharmed Gentili, Newron, and Teva, all of which are unrelated to this article. I. Berardelli disclosed that in the last 5 years, she has received consultant fees and/or lectures or has engaged in clinical trial activities with Angelini Pharma, Johnson & Johnson, Lundbeck, Rovi, all unrelated to this article. The authors have no other relevant affiliations or financial involvement with any organization or entity with a financial interest in or financial conflict with the subject matter or materials discussed in the manuscript apart from those disclosed.

## Data Availability

The data that support the findings of this study are available on request from the corresponding author. The data are not publicly available due to privacy or ethical restrictions.
